# Prevalence and psychopathology of vegetarians and vegans – Results from a representative survey in Germany

**DOI:** 10.1038/s41598-020-63910-y

**Published:** 2020-04-22

**Authors:** Georgios Paslakis, Candice Richardson, Mariel Nöhre, Elmar Brähler, Christina Holzapfel, Anja Hilbert, Martina de Zwaan

**Affiliations:** 1 Toronto General Hospital, University Health Network, Toronto, Canada; 2 Department of Psychiatry, University of Toronto, Toronto, Canada; 3 Department of Psychosomatic Medicine and Psychotherapy, Hannover Medical School, Hannover, Germany; 4 Department of Psychosomatic Medicine and Psychotherapy, University Medical Center Mainz, Mainz, Germany; 5 Institute for Nutritional Medicine, Else Kroener-Fresenius-Centre for Nutritional Medicine, School of Medicine, Klinikum rechts der Isar, Technical University of Munich, Munich, Germany; 6 Integrated Research and Treatment Center AdiposityDiseases, Behavioral Medicine Research Unit, Department of Psychosomatic Medicine and Psychotherapy, University of Leipzig Medical Center, Leipzig, Germany

**Keywords:** Psychology, Health care, Medical research

## Abstract

The aim of the study was to investigate the prevalence of, and attitudes toward, vegetarianism and veganism. We also assessed the association between vegetarianism/veganism and eating disorder, depressive, and somatic symptoms. A cross-sectional questionnaire survey in adults in Germany that was representative in terms of age, gender, and educational level was carried out. Data from 2449 adults (53.5% females) were included. Mean age was 49.6 (SD 17.1) years. A total of 5.4% of participants reported following a vegetarian or vegan diet. While the majority of participants agreed that vegetarian diets are healthy and harmless (56.1%), only 34.8% believed this to be true of vegan diets. The majority of participants also believed that a vegetarian (58.7%) or vegan (74.7%) diet can lead to nutritional deficiency. Female gender, younger age, higher education, lower body mass index (BMI), and higher depressive and eating disorder symptoms were found to be associated with vegetarianism/veganism. We did not find increased physical complaints in the group of vegetarians/vegans. Our results point toward a moderate prevalence of vegetarianism/veganism among the general population. Our findings suggest that health care professionals should keep eating disorder pathology, affective status in mind when dealing with individuals who choose a vegetarian/vegan dietary pattern.

## Introduction

To date research about vegetarianism and veganism is still young even though plant-based nutrition seems to have gained increasing popularity and represents a growing social movement^1^. Vegetarianism is a generic term that encompasses a variety of dietary patterns that each involves, to some extent, the avoidance of meat^2^. While omnivores consume all types of animal products^3^, true vegetarians are defined as those who do not eat any meat, poultry, or fish^4^. Vegetarians may be further sub-classified based on the inclusion of eggs (ovo-vegetarians), diary (lacto-vegetarians), fish (pesco-vegetarians), poultry (pollo-vegetarians), or a combination of these foods in their diet^2,5,6^. Finally, vegans are those who refrain from eating any animal products, including meat, fish, dairy, eggs, and other animal-derived foods^3^. Individuals may adopt a vegetarian diet for a variety of reasons which may be ethical, moral, religious, environmental, health-related, or concerns about animal welfare^7^. The majority of recent studies suggest that ethical concerns are the most common motivation for adopting a vegetarian diet, followed by health considerations^3,5,8^. While health vegetarians avoid meat in order to derive the perceived health benefits of a vegetarian diet or to lose weight, ethical vegetarians avoid meat for animal welfare reasons^9^. The aim of the present study was to investigate the prevalence of, and attitudes toward, vegetarianism and veganism in a representative sample of the general adult population in Germany.

The prevalence of vegetarianism varies around the world with recent polls indicating that approximately 5% of Americans^10^, 8% of Canadians^11^, and 4.3% of Germans^6^ follow a vegetarian diet. However, the highest proportion of vegetarians are found in India, where they comprise 30% of the population^12,13^. Veganism is less common with the prevalence reported to be about 2% in the United States^10^ and less than 1% in Germany^14^. In several studies, education and income were found to be inversely associated with meat consumption^14–16^. Subsequent research conducted within Central European countries also indicates that vegetarians tend to be more educated and affluent than omnivores^14,17^. Allès *et al*.^18^ confirmed that vegetarians tend to be more educated than omnivores, but also found out that vegans tend to have lower educational attainment. Additionally, women are far more likely to be vegetarian than men^8^; even among non-vegetarians, women have been found to eat considerably less meat than men^1,14,19,20^.

Although strict and unbalanced dietary restrictions can pose a risk of micronutrient deficiencies^2^, it is the position of the Academy of Nutrition and Dietetics that appropriately planned vegetarian diets are nutritionally adequate and may be beneficial for health^21^. A comprehensive meta-analysis of 80 studies provides evidence for the link between a vegetarian diet and a lowered risk of cardiovascular disease, type 2 diabetes, obesity, and certain types of cancer^22^. Vegetarianism has also been associated with reduced risk of hypertension, diverticular disease, degenerative arthritis, and metabolic syndrome in other studies^4,23^. Also, the use of vegetarian and vegan diets is often associated with other health behaviors including non-smoking and regular physical activity^1^. Additionally, studies conducted in Western countries have consistently shown that vegetarians have a lower BMI compared to their non-vegetarian counterparts, with vegans having the lowest BMI^24–26^.

There is mixed evidence as to whether vegetarianism is associated with more positive or negative mental health^27^. Although vegetarians have reported more positive mood in some studies^28,29^, they have described more psychological symptoms associated with anxiety and depression in others^30,31^. Similarly, while some studies^32–34^ have found a higher risk of depression among vegetarians, others^5,28^ show no significant difference between vegetarians and omnivores. Furthermore, adolescent vegetarians were more likely to be depressed^35^ and have contemplated suicide^36^ when compared to adolescent omnivores. Adherence to a vegetarian diet has been hypothesized to be a factor in the development and maintenance of disordered eating^5^ as the restrictive nature of the diet may be used as a socially acceptable way to refrain from eating specific foods^3^. The majority of studies^36–39^ suggest that vegetarians exhibit greater levels of disordered eating than omnivores; however, two recent studies suggest that this may not always be the case^40,41^. Indeed, in one study by Timko *et al*.^5^, semi-vegetarians, defined as those who exclude red meat from their diet, were found to have the highest level of eating pathology, while true vegetarians and vegans appeared healthiest in regards to eating and body weight. Thus, it is important to understand attitudes toward plant-based diets and the potential association with eating disorder, depressive, and somatic symptoms, as these findings, in conjunction with those from additional longitudinal studies, can lead to the development of more specific guidelines for healthcare professionals to monitor patients that follow these types of diets.

While the primary aim of this study was to investigate the prevalence of, and attitudes toward, vegetarianism and veganism, we also assessed the association between vegetarianism/veganism and eating disorder symptoms, depressive symptoms, and the presence of somatic symptoms. We expected that consistent with previous studies, vegetarians/vegans would display a higher burden of eating disorder and general psychopathology.

## Methods

### Recruitment

A random sample of German residents aged 14 years and older (age range 14 to 91 years) were recruited as part of a cross-sectional survey on physical and mental well-being. For the purposes of the present investigation we only assessed adults (≥18 years of age). A demographic consulting company (USUMA GmbH, Berlin, Germany) assisted with sampling and data collection. The procedure was designed to yield a nation-wide sample representative in terms of age, gender, and educational level over the fieldwork period from May to July 2018. Sociodemographic data were collected in-person by trained interviewers and participants also completed a battery of self-report questionnaires.

### Data acquisition

In Germany, no directory is generally available that contains the addresses of all private households or individuals, which could be used by market research agencies as a sampling frame. The data collected by the local authorities are only available for surveys considered to be of major public interest. A group of agencies called the “Arbeitsgemeinschaft ADM-Stichproben” closes this gap by providing a sampling frame to member agencies, the so-called “ADM-Sampling-System for Face-to-Face Surveys”. This frame allows representative face-to-face samples to be drawn for all households in Germany and for all people living in those households. In addition, the main statistical data are provided on a detailed level for this population. The ADM-Sampling-System is described in detail elsewhere^42,43^.

The participation rate was 46.9% (2531 of 5393 persons), taking into account all refusals to participate, as well as interviews that failed to take place due to respondents’ illness or being otherwise unavailable during the fieldwork. All participants provided their written informed consent in accordance with the Helsinki declaration. The study was approved by the Ethics Committee of the Medical School of the University of Leipzig.

The following sociodemographic data were assessed: gender (male and female), age (distinguished according to groups: 18–24, 25–34, 35–44, 45–54, 55–65, >65 years), educational level (<12 and ≥12 years), monthly income (0 to <1000, 1000–2500, and ≥2500 euros per month), population size (<5000, 5000–50000, and ≥50000 residents). The BMI was calculated based on participants’ self-reported height and weight.

### Dietary assessment

To assess self-reported dietary patterns, participants were asked the following question “Have you been consciously eating a vegetarian diet for at least 2 weeks?” This question has been repeated for vegan diet. It was explained to participants that vegetarian means omitting meat, but eating plants and milk products, and vegan means omitting all foods of animal origin.

Subsequently, participants were presented with a series of 11 statements about vegetarian diets. The same statements were asked for a vegan diet. Participants responded to each statement using a 4-point Likert scale ranging from “totally agree” to “totally disagree”. All statements were short and simple:

1. A vegetarian diet is completely healthy and harmless.
2. A vegetarian diet can lead to a nutritional deficiency.
3. People who follow a vegetarian diet are seldom overweight.
4. Individuals who follow a vegetarian diet are more productive.
5. A vegetarian diet is able to prevent disease.
6. A vegetarian diet is good for the environment.
7. A vegetarian diet is less cruel to animals.
8. People who follow a vegetarian diet are made fun of.
9. A vegetarian diet is not tasty.
10. A vegetarian diet is expensive.
11. People who eat a vegetarian diet do so out of ethical motivation.

Additionally, omnivores were asked if a vegetarian or vegan diet would be a viable diet for them to pursue. All questions and statements were constructed and finally chosen from a larger pool by experienced nutritionists, physicians and psychologists.

### Psychological assessment

Participants also completed the Patient Health Questionnaire-4 (PHQ-4)^44^, the Eating Disorder Examination-Questionnaire 8 (EDE-Q8)^45^, and a brief form of the Giessen Subjective Complaints List (GBB-8)^46^. The PHQ-4 allows for the brief measurement of depression and anxiety based on participants’ responses to 4 items on a Likert scale ranging from “not at all” to “nearly every day”. Total scores range from 0 to 12, and correspond to no (0–2), mild (3–5), moderate (6–8), or severe (9–12) psychological distress^44^. The EDE-Q8 is an 8-item self-report questionnaire used to assess eating disorder psychopathology. Scores range from 0 to 6, with higher scores indicating greater psychopathology^45^. Similarly, the GBB-8 is a brief, self-report questionnaire used to assess somatic symptom strain. Total scores range from 0 to 32, while scores on the four subscales (exhaustion, gastrointestinal complaints, musculoskeletal complaints, and cardiovascular complaints) range from 0 to 8 with higher scores indicating greater symptom strain^46^.

### Statistical analyses

For analysis of the dietary statements, the answers “totally agree” and “agree” were grouped together, as were “disagree” and “totally disagree”. T-Tests or Chi-square tests were performed appropriately when comparing vegetarians/vegans with omnivores. In order to examine the predictive value of the independent variables, a binary logistic regression was performed with vegetarianism and veganism as dependent variable. The level of significance was set at p ≤ 0.05. Bonferroni correction for multiple testing was performed according to the number of independent variables in each hypothesis testing. Unweighted data were used. Statistical analyses were performed using SPSS (IBM SPSS Statistics for Windows, Version 25.0. Armonk, NY: IBM Corp.).

## Results

### Participants

A total of 2531 individuals participated in the survey. Of those, 82 were excluded for being younger than 18 years. Thus, data from a total of 2449 adults were analyzed. This cohort consisted of 46.5% (1138/2449) males and 53.5% (1311/2449) females. Mean age was 49.6 (SD 17.1) years, and 40.9% (1001/2449) of participants were aged older than 55 years. Additionally, the mean BMI was 25.9 (SD 2.1) kg/m^2^. More details on socio-demographics of the cohort are shown in Table 1.

**Table 1:** Socio-demographic characteristics of survey participants.

Variables	Survey participants
	N (%)
Gender	
Male	1138 (46.5%)
Female	1311 (53.5%)
	2449 (100%)
Age groups [years]	
18–24	198 (8.1%)
25–34	378 (15.4%)
35–44	397 (16.2%)
45–54	475 (18.7%)
55–64	458 (18.7%)
65+	543 (22.2%)
	2449 (100%)
Education [years]	
<12	1873 (76.5%)
≥12	572 (23.4%)
	2445 (99.8%)
Marital status	
Married, living with spouse	1101 (45.0%)
Married, living separate from spouse	70 (2.9%)
Single	679 (27.7%)
Divorced	368 (15.0%)
Widowed	220 (9.0%)
	2438 (99.6%)
Income groups [Euro/month]	
0 to <1000	585 (23.9%)
1000 to <2500	1523 (62.2%)
≥2500	262 (10.7%)
	2370 (96.8%)
Population size	
<5000	425 (17.4%)
5000 to <50000	935 (38.2%)
≥50000	1089 (44.5%)
	2449 (100%)

### Prevalence of self-defined vegetarianism and veganism

When participants were asked whether they have followed a conscious vegetarian diet for at least two weeks, 5.2% (126/2444) indicated yes. The non-vegetarian participants were further asked whether a vegetarian diet would be a viable diet form to pursue. Of the 2316 respondents to this question, 11.2% (259/2316) indicated yes. In a similar manner, 1.3% (31/2446) of participants reported following a strict vegan diet. The non-vegan participants were further asked whether a vegan diet would be a potential diet form to adopt, to which 5.9% (142/2414) indicated yes.

There was considerable overlap in the endorsement of vegetarian and vegan dietary patterns. Of the 133 (5.4%) participants who reported following a vegetarian and/or vegan diet, 18% (24/133) answered “yes” to both questions, 76.7% (102/133) reported following a vegetarian but not a vegan diet, and 5.3% (7/133) indicated they followed a vegan but not vegetarian diet. Given this overlap, for the present analysis, those who endorsed a vegetarian and/or vegan diet were grouped together. Thus, the prevalence of current self-defined vegetarians/vegans was 5.4% in the present sample.

### Attitudes toward vegetarian and vegan diets

When participants were asked about their attitudes toward a vegetarian diet, the majority of both vegetarians/vegans and omnivores agreed that a vegetarian diet is completely healthy and harmless, good for the environment, and less cruel to animals. The majority of both groups also believed that those who eat a vegetarian diet do so out of ethical motivation, and are seldom overweight. Conversely, the majority of both groups disagreed with the notions that vegetarians are made fun of and that a vegetarian diet is not tasty. While the majority of vegetarians/vegans agreed that vegetarians are more productive and that the diet can prevent disease, only a minority of omnivores agreed with these statements. Similarly, while the majority of omnivores agreed that a vegetarian diet is expensive and can lead to nutritional deficiencies, the majority of vegetarians/vegans disagreed with these statements. Full details are displayed in Table 2.

**Table 2:** Participants’ attitudes toward vegetarian diets.

	Vegetarians/Vegans			Omnivores			Total			χ^2^ p
	Agree n (%)	Disagree n (%)	N	Agree n (%)	Disagree n (%)	N	Agree n (%)	Disagree n (%)	N	
A vegetarian diet is completely healthy and harmless	128 (97.0%)	4 (3.0%)	132	1232 (53.7%)	1063 (46.3%)	2295	1360 (56.0%)	1067 (44.0%)	2427	94.94 <0.001
A vegetarian diet can lead to a nutritional deficiency	34 (25.8%)	98 (74.2%)	132	1398 (60.6%)	908 (39.4%)	2306	1432 (58.7%)	1006 (41.3%)	2438	62.63 <0.001
People who follow a vegetarian diet are seldom overweight	98 (73.7%)	35 (26.3%)	133	1495 (64.8%)	811 (35.2%)	2306	1593 (65.3%)	846 (34.7%)	2439	4.35 0.022
Individuals who follow a vegetarian diet are more productive	105 (78.9%)	28 (21.1%)	133	590 (25.6%)	1712 (74.4%)	2302	695 (28.5%)	1740 (71.5%)	2435	175.25 <0.001
A vegetarian diet is able to prevent disease	111 (83.5%)	22 (16.5%)	133	815 (35.5%)	1483 (64.5%)	2298	926 (38.1%)	1505 (61.9%)	2431	122.80 <0.001
A vegetarian diet is good for the environment	123 (92.5%)	10 (7.5%)	133	1161 (50.5%)	1139 (49.5%)	2300	1284 (52.8%)	1149 (47.2%)	2433	89.00 <0.001
A vegetarian diet is less cruel to animals	117 (88.0%)	16 (12.0%)	133	1434 (62.3%)	867 (37.7%)	2301	1551 (63.7%)	883 (36.3%)	2434	35.78 <0.001
People who follow a vegetarian diet are made fun of	63 (47.4%)	70 (52.6%)	133	1117 (48.7%)	1175 (51.3%)	2292	1180 (48.7%)	1245 (51.3%)	2425	0.09 0.414
A vegetarian diet is not tasty	15 (11.4%)	117 (88.6%)	132	1101 (48.0%)	1195 (52.0%)	2296	1116 (46.0%)	1312 (54.0%)	2428	67.28 <0.001
A vegetarian diet is expensive	36 (27.1%)	97 (72.9%)	133	1403 (61.1%)	895 (38.9%)	2298	1439 (59.2%)	992 (40.8%)	2431	60.12 <0.001
People who eat a vegetarian diet do so out of ethical motivation	98 (74.2%)	34 (25.8%)	132	1579 (69.0%)	709 (31.0%)	2288	1677 (69.3%)	743 (30.7%)	2420	1.61 0.120

Similarly, when asked about their attitudes toward a vegan diet, the majority of vegetarians/vegans and omnivores, again, agreed that those who eat a vegan diet do so out of ethical motivation, are seldom overweight, and that a vegan diet is good for the environment and less cruel toward animals. However, in opposition to their attitudes toward vegetarian diets, the majority of both groups believed that a vegan diet can lead to nutritional deficiency, and that those who follow a vegan diet are made fun of. While the majority of vegetarians/vegans agreed that a vegan diet is completely healthy and harmless, can prevent disease, and that those who follow a vegan diet are more productive, the majority of omnivores disagreed with these statements. Similarly, while the majority of omnivores agreed that a vegan diet is expensive and not tasty, the majority of vegetarians/vegans disagreed with these notions. Full details are displayed in Table 3.

**Table 3:** Participants’ attitudes toward vegan diets.

	Vegetarians/Vegans			Omnivores			Total			χ^2^ p
	Agree n (%)	Disagree n (%)	N	Agree n (%)	Disagree n (%)	N	Agree n (%)	Disagree n (%)	N	
A vegan diet is completely healthy and harmless	90 (67.7%)	43 (32.3%)	133	752 (32.8%)	1538 (67.2%)	2290	842 (34.8%)	1581 (65.2%)	2423	67.26 <0.001
A vegan diet can lead to a nutritional deficiency	80 (60.2%)	53 (39.8%)	133	1735 (75.5%)	563 (24.5%)	2298	1815 (74.7%)	616 (25.3%)	2431	15.66 <0.001
People who follow a vegan diet are seldom overweight	109 (82.0%)	24 (18.0%)	133	1531 (66.6%)	767 (33.4%)	2298	1640 (67.5%)	791 (32.5%)	2431	13.46 <0.001
Individuals who follow a vegan diet are more productive	73 (55.3%)	59 (44.7%)	132	455 (19.8%)	1838 (80.2%)	2293	528 (21.8%)	1897 (78.2%)	2425	92.14 <0.001
A vegan diet is able to prevent disease	85 (63.9%)	48 (36.1%)	133	671 (29.3%)	1618 (70.7%)	2289	756 (31.2%)	1666 (68.8%)	2422	70.07 <0.001
A vegan diet is good for the environment	114 (85.7%)	19 (14.3%)	133	1172 (51.2%)	1119 (48.8%)	2291	1286 (53.1%)	1138 (46.9%)	2424	60.27 <0.001
A vegan diet is less cruel to animals	114 (85.7%)	19 (14.3%)	133	1441 (62.8%)	854 (37.2%)	2295	1555 (64.0%)	873 (36.0%)	2428	28.69 <0.001
People who follow a vegan diet are made fun of	95 (73.1%)	35 (26.9%)	130	1469 (64.1%)	821 (35.9%)	2290	1564 (64.6%)	856 (35.4%)	2420	4.29 0.022
A vegan diet is not tasty	28 (21.2%)	104 (78.8%)	132	1461 (63.9%)	825 (36.1%)	2286	1489 (61.6%)	929 (38.4%)	2418	96.167 <0.001
A vegan diet is expensive	63 (47.4%)	70 (52.6%)	133	1675 (73.2%)	614 (26.8%)	2289	1738 (71.8%)	684 (28.2%)	2422	41.311 <0.001
People who eat a vegan diet do so out of ethical motivation	106 (79.7%)	27 (20.3%)	133	1670 (73.1%)	614 (26.9%)	2284	1776 (73.5%)	641 (26.5%)	2417	2.794 0.055

### Comparison between self-defined vegetarians/vegans and omnivores

Comparing vegetarians/vegans to omnivores, no differences in income distribution or population size of the community or city of origin were found. Among vegetarians/vegans, a significantly higher proportion were female (73.7% vs. 26.3%; X^2^(1) = 23.174, p < 0.001). Additionally, vegetarians/vegans were significantly younger than omnivores (M = 40.9, SD = 15.5 vs. M = 50.0, SD = 17.0; t(2442) = −6.033, p < 0.001). Finally, 51.5% of vegetarians/vegans attained 12 or more years of education compared to 21.8% of omnivores (X^2^(1) = 61.531, p < 0.001). Vegetarians/vegans also had a significantly lower BMI compared to omnivores (M = 24.0, SD = 4.7 vs. M = 26.0, SD = 5.0; t(2423) = −4.555, p < 0.001). Similarly, vegetarians/vegans had significantly higher eating disorder psychopathology in the EDE-Q8 (M = 1.3, SD = 1.4 vs. M = 1.0, SD = 1.3; t(2440) = 2.619, p = 0.009), as well as slightly, but not significantly, higher depression scores in the PHQ-4 (M = 2.0, SD = 2.3 vs. M = 1.5, SD = 2.1; t(140) = 2.327, p = 0.21) scores compared to omnivores. More details are shown in Table 4. In terms of somatic complaints, vegetarians/vegans and omnivores did not significantly differ in their experiences of exhaustion, gastrointestinal complaints, musculoskeletal complaints, cardiovascular complaints, or overall symptom strain (Table 5).

**Table 4:** Comparison between vegetarians/vegans and omnivores.

	Vegetarians/Vegans	Omnivores	Statistics
**Gender, n (%)**			
Male	35 (26.3%)	1103 (47.7%)	X^2^(1) = 23.174, p < 0.001
Female	98 (73.7%)	1208 (52.3%)	
	133 (100%)	2311 (100%)	
**Age groups [years], n (%)**			
18–24	21 (15.8%)	176 (7.6%)	
25–34	38 (28.6%)	340 (14.7%)	
35–44	15 (11.3%)	380 (16.4%)	X^2^(5) = 49.788, p < 0.001
45–54	33 (24.8%)	442 (19.1%)	
55–64	19 (14.3%)	439 (19.0%)	
65+	7 (5.3%)	534 (23.1%)	
	133 (100%)	2311 (100%)	
**Education [years], n (%)**			
<12	64 (48.5%)	1805 (78.2%)	X^2^(1) = 61.531, p < 0.001
≥12	68 (51.5%)	503 (21.8%)	
	132 (100%)	2308 (100%)	
**Income groups [Euro/month], n(%)**			
0 to <1000	33 (25.6%)	550 (24.6%)	
1000 to <2500	85 (65.9%)	1436 (64.2%)	X^2^(2) = 0.903 p = 0.64
≥2500	11 (8.5%)	251 (11.2%)	
	129 (100%)	2237 (100%)	
**Population size, n (%)**			
<5000	16 (12.0%)	408 (17.7%)	
5000 to <50000	48 (36.1%)	886 (38.3%)	X^2^(2) = 4.214, p = 0.12
≥50000	69 (51.9%)	1017 (44.0%)	
	133 (100%)	2311 (100%)	
**BMI, n (%)**			
BMI < 30 kg/m^2^	121 (91.7%)	1943 (84.7%)	X^2^(1) = 4.732, p = 0.03
BMI ≥ 30 kg/m^2^	11 (8.3%)	350 (15.3%)	
BMI, mean (SD)	24.0 (4.7)	26.1 (5.0)	t(2423) = −4.555, p < 0.001
**EDE-Q8, mean (SD)**	1.3 (1.4)	1.0 (1.3)	t(2440) = 2.619, p = 0.01
**PHQ-4, n(%)**			
<6	118 (91.5%)	2154 (94.7%)	X^2^(1) = 2.502, *P* = 0.112
≥6	11 (8.5%)	120 (5.3%)	
PHQ-4, mean (SD)	2.02 (2.31)	1.54 (2.11)	t(140) = 2.327, p = 0.21

BMI: Body Mass Index, PHQ-4: Patient Health Questionnaire, EDE-Q8: Eating Disorder Examination Questionnaire.

**Table 5:** Somatic symptoms (GBB-8) of vegetarians/vegans and omnivores.

Variables	Vegetarians/Vegans mean (SD)	Omnivores Mean (SD)	Statistics
n	133	2311	
Total	4.8	4.5	t = 0.656 p = 0.51
Exhaustion	1.8	1.6	t = 1.048 p = 0.30
Gastrointestinal complaints	1.0	0.7	t = 1.467 p = 0.15
Musculoskeletal complaints	1.6	1.6	t = −0.333 p = 0.74
Cardiovascular complaints	0.7	0.6	t = 0.554 p = 0.58

GBB-8: Giessen Subjective Complaint List.

A binary logistic regression analysis was conducted to predict vegetarianism/veganism based on gender, age, education, population size, income, BMI, EDE-Q8 score, and PHQ-4 score. A significant regression model was found, χ^2^(8) = 835.0, p < 0.001: gender (female), (younger) age, (higher) education, (lower) BMI, (higher) PHQ-4 score, and (higher) EDE-Q8 score were significant statistical predictors of vegetarianism/veganism (Table 6).

**Table 6:** Results of the binary logistic regression analysis to predict vegetarianism/veganism based on gender, age, education level, population size, income, BMI, PHQ-4 score, and EDE-Q8 score.

	OR	Standard error	p	95% confidence interval	
				Lower value	Upper value
Constant	3.437	0.845	0.144		
Gender	1.028	0.236	**<0.001**	0.248	0.625
Age	0.359	0.007	**<0.001**	1.015	1.041
Educational level	0.843	0.201	**<0.001**	0.242	0.533
Population size	0.849	0.141	0.224	0.639	1.110
Income	1.102	0.175	0.347	0.603	1.195
BMI	0.901	0.028	**<0.001**	1.043	1.163
PHQ-4 score	0.834	0.043	**0.016**	0.829	0.981
EDE-Q8 score	3.437	0.082	**0.027**	0.711	0.979

BMI: Body Mass Index, PHQ-4: Patient Health Questionnaire, EDE-Q8: Eating Disorder Examination Questionnaire.

## Discussion

The prevalence rate of self-defined vegetarians/vegans among the general German population found in the present investigation (n = 133, 5.4%) is comparable to that reported in an earlier German representative sample, in which the prevalence was found to be between 3% and 6%^14^. The current prevalence rate is also similar to those reported in US investigations^10^. A significant minority of omnivores reported that they would consider a vegetarian (11.2%) or vegan (5.9%) diet for themselves in the future showing that some people have an interest in adopting this kind of nutrition (“prospective vegetarianism”)^20^. Omnivores were more open to becoming vegetarian than to becoming vegan. However, meat consumption is still part of the traditional and social norm in Western societies^47^.

Regarding general attitudes toward vegetarian/vegan forms of diet in the general population, we noticed that both diet forms were considered expensive. Vegetarian and vegan diets are often perceived to be expensive^48^, and have therefore been associated with lower openness to try a vegetarian diet^49^. However, when compared to meat eaters, “true” vegetarians have been shown to report lower food expenditures^50^. While a vegetarian diet was considered to be healthy by most respondents, the majority did not think the same about vegan diets. This is in line with the German Nutrition Society which clearly states that a vegan diet cannot fulfill the daily recommendation for vitamin B12 intake and that supplementation is needed in most of the persons sticking on a vegan diet. Moreover, veganism is not recommended for pregnant and lactating women^51^. Additionally, almost two-third of the respondents said that vegans are made fun of and also a slight majority of respondents agreed that vegetarians are made fun of. Most respondents stated that vegetarian/vegan diet is less often associated with overweight. Vegan diet is not considered tasty. People have more negative beliefs about veganism than vegetarianism which is in line with literature. Literature also shows that vegetarians themselves report unfavorable social experiences^52^ and biases with omnivores belittling their character^20^.

We found that female gender, younger age, lower BMI, higher depression scores, and higher eating disorder-related psychopathology were significantly associated with vegetarian/vegan diets. In terms of gender differences, this is entirely in accordance with the existing literature^1,8,14^. There is an extensive literature on the association between meat and masculinity^19^ showing that men view meat as a more essential part of a proper diet. Thus it is not surprising that we found a higher proportion of females to be vegetarians/vegans. Similarly, we found that vegetarians/vegans were significantly younger than omnivores, as previously documented in multiple studies conducted among adults in Germany^14^, the UK, Canada, and the U.S^18,24,53–55^. Our findings are also in congruence with previous research showing an association between higher education and reduced meat consumption^14–16^, as over half of the vegetarians/vegans in our study attained 12 or more years of education compared to 22% of omnivores. Conversely, while previous research has documented greater affluence among vegetarians^14,17^, we found no difference in income distribution between vegetarians/vegans and omnivores in our sample. We also found vegetarians/vegans to have a lower BMI compared to their omnivorous counterparts; which is consistent with existing literature^10,24–26^.

Limited data is available on the associations between vegetarian diet and mental health^34^. While some studies have shown no significant differences in depressive symptoms between vegetarians and omnivores^5,28^, our results more closely align with those that have documented higher risk for^32–34^ and more psychological symptoms associated with depression^30,31^ among vegetarians/vegans. The prevalence of participants who screened positive for potential cases of depression and anxiety (PHQ-4 > 6) was 5.3% in omnivores and 8.5% in vegetarians/vegans. However, we cannot make any assumptions about causality. Do more depressed individuals select to follow a vegetarian/vegan diet or does following a vegetarian/vegan diet increase the risk for developing depression? It cannot be excluded that nutritional status may affect brain processes and may influence onset and maintenance of mental disorders^34^.

Our results allude to an association between choosing to subsist upon a diet excluding meat and displaying symptoms of disordered eating. The difference in EDE-Q8 scores remained even after adjusting for gender and age which are known to influence eating disorder symptoms. This result is in accordance to the majority of previous similar studies^36–39^, even though the overall scores in the present sample were close to scores found in the general German population^45^. In terms of the potential link between vegetarianism and the development of eating disorders, evidence from three retrospective chart reviews^56–58^ show that approximately half of all patients diagnosed with anorexia nervosa report adhering to a vegetarian diet. Others have emphasized that this might represent a more orthorexic behavior with a fixation on health-conscious eating^59^. Furthermore, two-thirds of individuals with history of an eating disorder reported that their vegetarianism was related to the eating disorder as it allowed them to restrict caloric intake and increase feelings of control; however, the majority of these individuals also indicated that they adopted a vegetarian diet after the onset of their eating disorder^60^. Thus, vegetarianism may be a symptom of the disorder or a maintaining factor, rather than linked to its causal development^3^. As far as the clinical implications of our findings are concerned, our findings imply that health care professionals should keep the association between eating disorder psychopathology and vegetarian/vegan forms of diet in mind when dealing with individuals who choose this form of diet; especially in younger women. Similarly, affective status should be considered in the same group in question.

We did not find differences with regard to physical complaints between the groups of vegetarians/vegans and omnivores. Thus, although self-reported symptoms cannot be accounted for factual differences in health status between the two groups under investigation, we conclude that vegetarians did not differ in complaints of (somatoform) symptoms compared to the general omnivore population. In one German study vegetarians reported a better current health status than omnivores^14^. This finding is in contrast to another previous German investigation showing an increased prevalence for somatoform disorders in vegetarians^34^. Michalak *et al*.^34^ found evidence for elevated prevalence rates in vegetarians not only for somatoform syndromes but also for depressive disorders, anxiety disorders as well as for eating disorders. This is the only study that did not rely on self-report but used clinical diagnoses of mental disorders as assessed with standardized diagnostic interviews and that controlled for socio-demographic characteristics. For depressive, anxiety and somatoform disorders the adoption of a vegetarian diet followed the onset of mental disorders and the authors hypothesized that a mental disorder increases the likelihood of choosing a vegetarian diet probably with the goal to positively influence the course of the disease.

There are some limitations to consider. The response rate was relatively low (46.9%), which is, however, common in general population research. The current use of vegetarian and vegan diets was self-reported, and it is known from previous studies that self-identified vegetarians do not necessarily completely abstain from meat^6,19^. Furthermore, we define “vegetarian/vegan” as a person who sticks to that diet for at least two weeks, which means, that this definition is rather lenient. Therefore, also short-term vegetarians/vegans might be within the survey. We did not assess the motivation for following a vegetarian or vegan diet. A further limitation lies in the lack of assessment of objective measures (e.g., BMI) and the reliance on self-reports. Additionally, considering that this was a cross-sectional study, and that the vegetarian/vegan group was mostly young females, it may be that young females are more prone to having an eating disorder regardless of the diet they follow. Thus, vegetarianism/veganism may be a symptom or maintaining factor of the disorder rather than linked to its causal development. Due to the cross-sectional design in this study, no conclusions can be made regarding the causality of the association between diet and the examined individual differences. In contrast, the strengths of our study are the inclusion of a large representative sample of the German population and the use of standardized questionnaires to assess depressive, eating disorder, and somatic symptoms.

Taken together, the prevalence of current and self-defined vegetarianism and veganism in the present research was 5.4% which is comparable to other German and international studies. People’s attitudes toward vegetarians and vegans still point toward some biases. Finally, the present survey showed that there are not only differences between self-defined vegetarians and omnivores in socio-demographics, but also in levels of eating-related symptoms and potential cases of depression and anxiety.

## Data Availability

The datasets generated during and/or analyzed during the current study are available from the corresponding author on reasonable request.
