# Author Correction: Prevalence and psychopathology of vegetarians and vegans – Results from a representative survey in Germany

**DOI:** 10.1038/s41598-020-76733-8

**Published:** 2020-11-10

**Authors:** Georgios Paslakis, Candice Richardson, Mariel Nöhre, Elmar Brähler, Christina Holzapfel, Anja Hilbert, Martina de Zwaan

**Affiliations:** 1 Toronto General Hospital, University Health Network, Toronto, Canada; 2 Department of Psychiatry, University of Toronto, Toronto, Canada; 3 Department of Psychosomatic Medicine and Psychotherapy, Hannover Medical School, Hannover, Germany; 4 Department of Psychosomatic Medicine and Psychotherapy, University Medical Center Mainz, Mainz, Germany; 5 Institute for Nutritional Medicine, Else Kroener-Fresenius-Centre for Nutritional Medicine, School of Medicine, Klinikum Rechts Der Isar, Technical University of Munich, Munich, Germany; 6 Integrated Research and Treatment Center AdiposityDiseases, Behavioral Medicine Research Unit, Department of Psychosomatic Medicine and Psychotherapy, University of Leipzig Medical Center, Leipzig, Germany

Correction to: *Scientific Reports* 10.1038/s41598-020-63910-y, published online 22 April 2020

This Article contains an error in the p-value reported for scores in the Patient Health Questionnaire-4 (PHQ-4).

As a result, in the Results section, under the subheading ‘Comparison between self-defined vegetarians/vegans and omnivores’,

“Similarly, vegetarians/vegans had significantly higher eating disorder psychopathology in the EDE-Q8 (M = 1.3, SD = 1.4 vs. M = 1.0, SD = 1.3; t(2440) = 2.619, p = 0.009), as well as slightly, but not significantly, higher depression scores in the PHQ-4 (M = 2.0, SD = 2.3 vs. M = 1.5, SD = 2.1; t(140) = 2.327, p = 0.21) scores compared to omnivores”

should read:

“Similarly, vegetarians/vegans had significantly higher eating disorder psychopathology in the EDE-Q8 (M = 1.3, SD = 1.4 vs. M = 1.0, SD = 1.3; t(2440) = 2.619, p = 0.009), as well as significantly higher scores in the PHQ-4 (M = 2.0, SD = 2.3 vs. M = 1.5, SD = 2.1; t(140) = 2.327, p = 0.021) compared to omnivores”

Furthermore, the incorrect p-value is also reported in Table 4. In the ‘Statistics’ column for row ‘PHQ-4, mean (SD)’,

“p = 0.21”

should read:

“p = 0.021”

